# Supplementary material for: Highly parallel and ultra-low-power probabilistic reasoning with programmable gaussian-like memory transistors
Source: Nat Commun. 2024 Mar 18;15:2439. doi: 10.1038/s41467-024-46681-2 (PMC10948914; doi:10.1038/s41467-024-46681-2)
Supplement: Supplementary file 1 — Supplementary Information [file 41467_2024_46681_MOESM1_ESM.pdf]

Supplementary Information

Highly Parallel and Ultra-Low-Power Probabilistic Reasoning with Programmable Gaussian-like Memory Transistors

Changhyeon Lee,[1,†] *Leila Rahimifard,*[2,†] *Junhwan Choi,*[3,†] *Jeong-ik Park,* [1] *Chungryeol Lee,*[1] *Divake Kumar,*[2] *Priyesh Shukla,*[2], *Seung Min Lee,*[1] *Amit Ranjan Trivedi,*[*,2]*Hocheon Yoo,*[*,4] and *Sung Gap Im*[*,1,5]

[1] *Department of Chemical and Biomolecular Engineering*
Korea Advanced Institute of Science and Technology (KAIST)
291 Daehak-ro, Yuseong-gu, Daejeon, 34141, Korea

[2] *Department of Electrical and Computer Engineering*
University of Illinois at Chicago
Chicago, IL 60607 USA

[3]*Department of Chemical Engineering*
Dankook University
152 Jukjeon-ro, Suji-gu, Yongin, Gyeonggi-do, 16890, Korea

[4]*Department of Electronic Engineering*
Gachon University
1342 Seongnam-daero, Sujeong-gu, Seongnam, Gyeonggi-do, 13120, Korea

[5] *KAIST Institute For NanoCentury (KINC)*
Korea Advanced Institute of Science and Technology (KAIST)
291 Daehak-ro, Yuseong-gu, Daejeon, 34141, Korea

[†]These authors contributed equally to this work.

Correspondence and requests for materials should be addressed to A.T.(amitrt@uic.edu), H.Y. (hyoo@gachon.ac.kr) or S.G.I. (sgim@kaist.ac.kr).

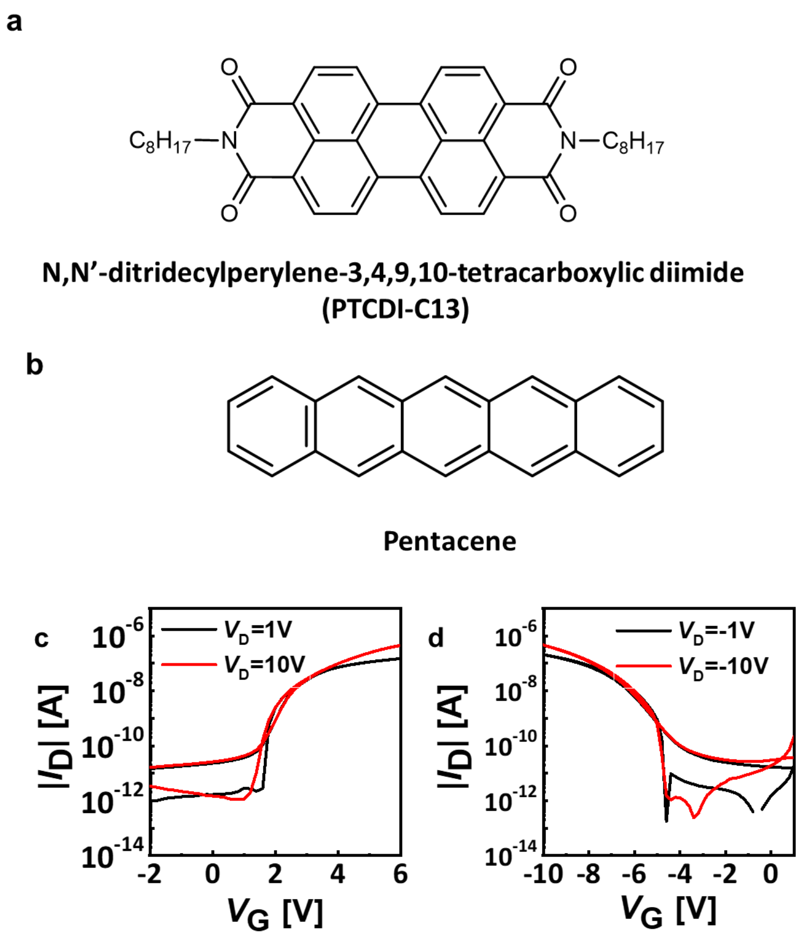

Supplementary Fig. 1 : Chemical structure and electrical performance of semiconductor.

a,b, The chemical structures of the n-type organic semiconductors (PTCDI-C13) (**a**) and p-type semiconductor (pentacene) (**b**), used in this study. **c,d,** Transfer characteristics (absolute value of drain current (|I_D|) verse gate voltage (V_G)) of unit n-type (**c**) and p-type (**d**) transistor.

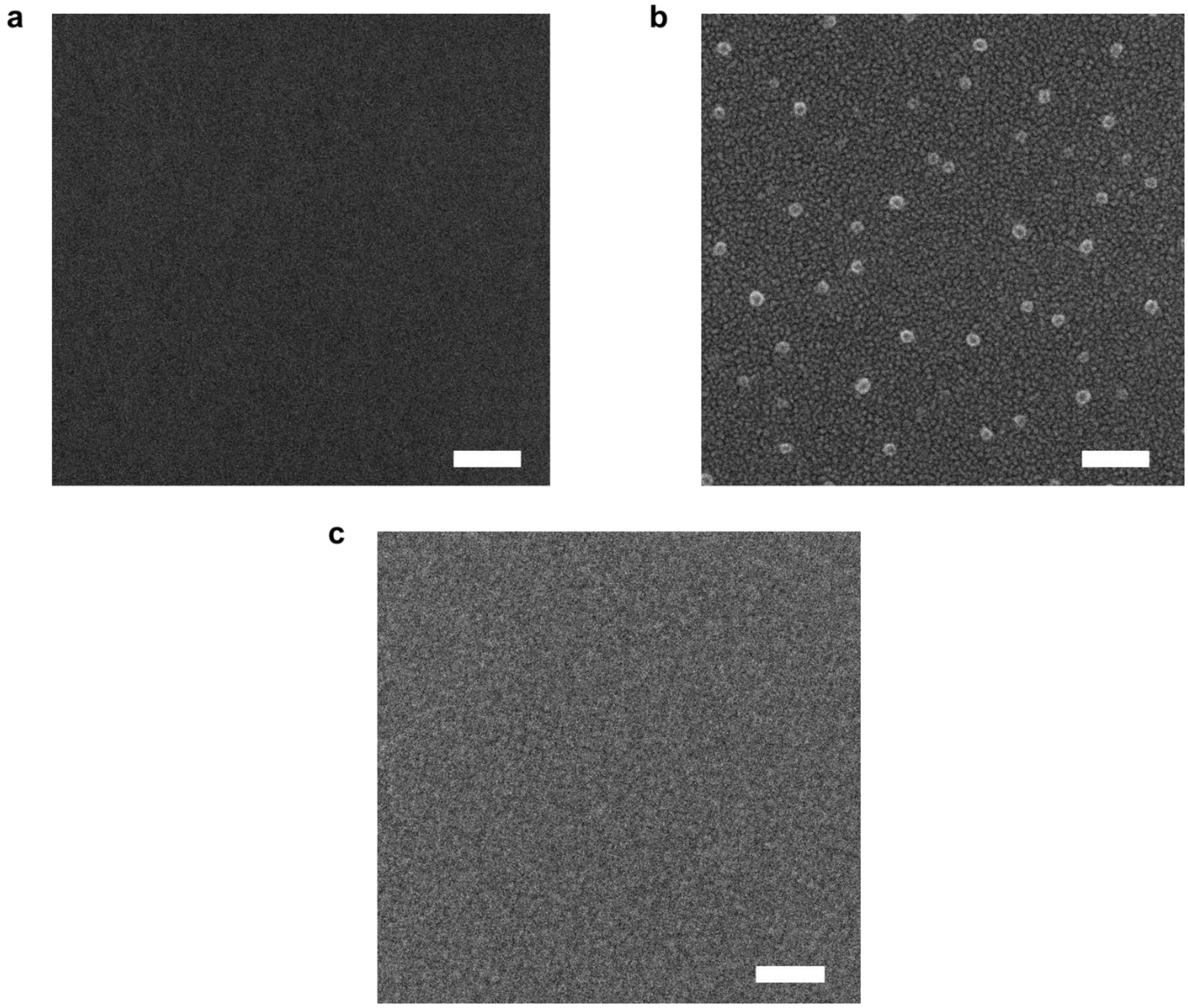

Supplementary Fig. 2 : Scanning electron microscope images

a,b,c, The scanning electron microscope (SEM) images show the C1D1 dielectric before deposition of Gold nanoparticle (AuNP) (a), after the deposition of AuNP on poly(2-cyanoethyl acrylate-co-diethylene glycol divinyl ether) [p(CEA-co-DEGDVE)] (pC1D1) dielectric (b), and the poly(1,3,5-trivinyl-1,3,5-trimethyl cyclotrisiloxane) (pV3D3) layer on AuNP on pC1D1 dielectric (c) (scale bar = 100 nm).

Material design and basic operation of GMT device

For the dielectric layers, polymer dielectric materials deposited via an initiated chemical vapor deposition (iCVD) process was used to ensure the superb insulating properties even with ultrathin thickness and mechanical flexibility as well as to provide the proper interface to the organic semiconductors [1, 2, 3, 4, 5, 6]. The high-*k* poly(2-cyanoethyl acrylate-co-diethylene glycol divinyl ether) [p(CEA-co-DEGDVE)] with the optimized chemical composition (named pC1D1) was used as a blocking dielectric layer (BDL)[1, 6], and low-*k* poly(1,3,5-trivinyl-1,3,5-trimethyl cyclotrisiloxane) (pV3D3) (14 nm) was used as a tunneling dielectric layer (TDL). The iCVD process allowed the thickness of the pV3D3 TDL to be low (~14 nm) while maintaining robust dielectric properties, leading to the low operating voltage [1, 2, 4]. Moreover, the high dielectric constant of pC1D1 BDL (k ~ 6.2) and low dielectric constant of pV3D3 TDL (k ~ 2.2) makes the GMT device have high gate coupling ratio ($\alpha_{CR}=V_{FG}/V_{G}$, where V_{FG} is the amount of voltage applied to FG)[1, 3]. Therefore, Fowler-Nordheim (F-N)-like tunneling was promoted according to the higher electric field (E) applied to the TDL. The high α_{CR} is also advantageous for the BDL by reducing the E applied to the BDL, resulting in suppression of the current leakage through BDL. In addition, the high dielectric constant of the pC1D1 BDL enabled low voltage operation even with relatively thick (~100 nm) thickness. (Supplementary Fig. 3)

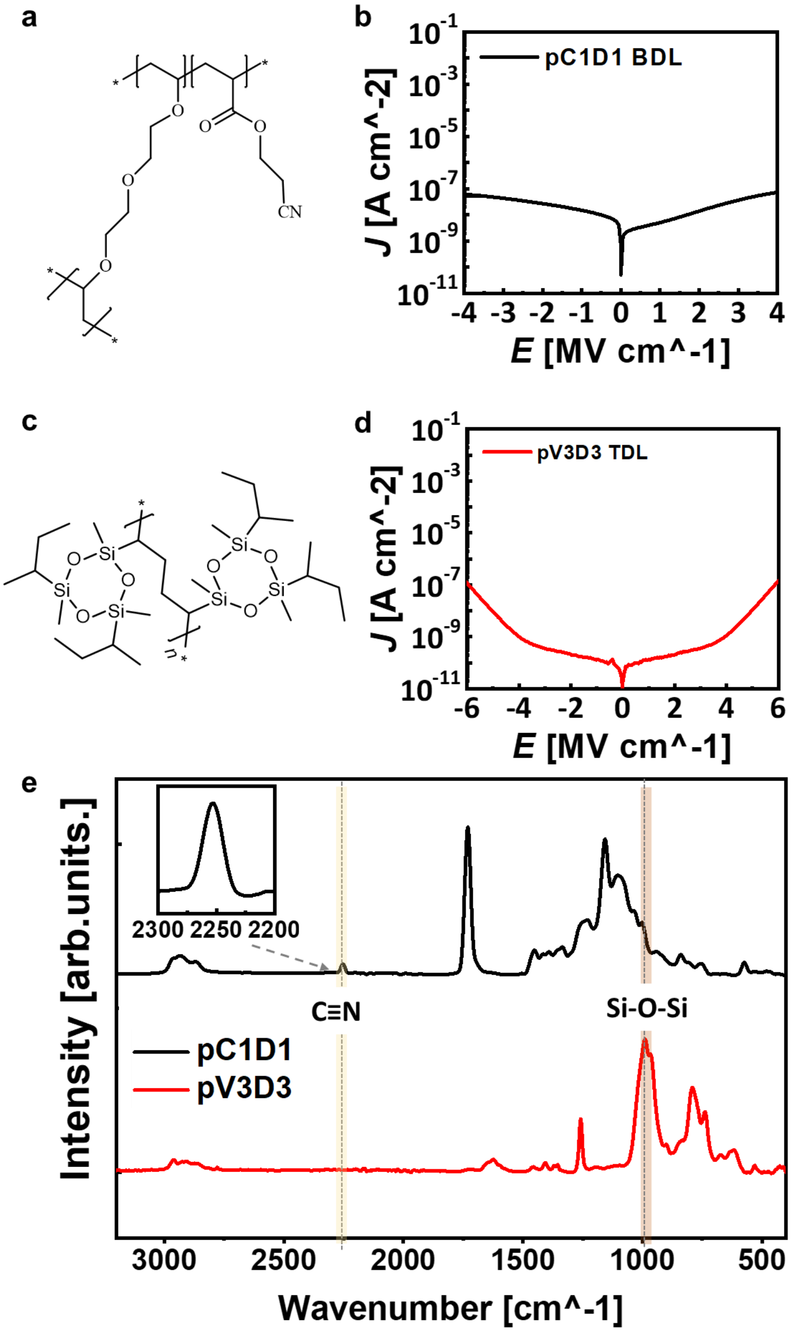

Supplementary Fig. 3: Material characterization of polymer dielectrics

a,b, The chemical structure of poly(2-cyanoethyl acrylate-co-diethylene glycol divinyl ether) [p(CEA-co-DEGDVE)] (pC1D1) used as blocking dielectric layer(BDL) (**a**) and absolute value of current density ($|J_i|$) vs electric field (E_i) characteristics of a Al/pC1D1 (100 nm)/Al device (**b**). **c,d,** The chemical structures of the poly(1,3,5-trivinyl-1,3,5-trimethyl cyclotrisiloxane) (pV3D3) polymer dielectrics (**c**) and $|J_i|$ vs E_i characteristics of a Al/pV3D3 (14 nm)/Al device (**d**). **e,** FT-IR spectra of the pC1D1 and pV3D3 films.

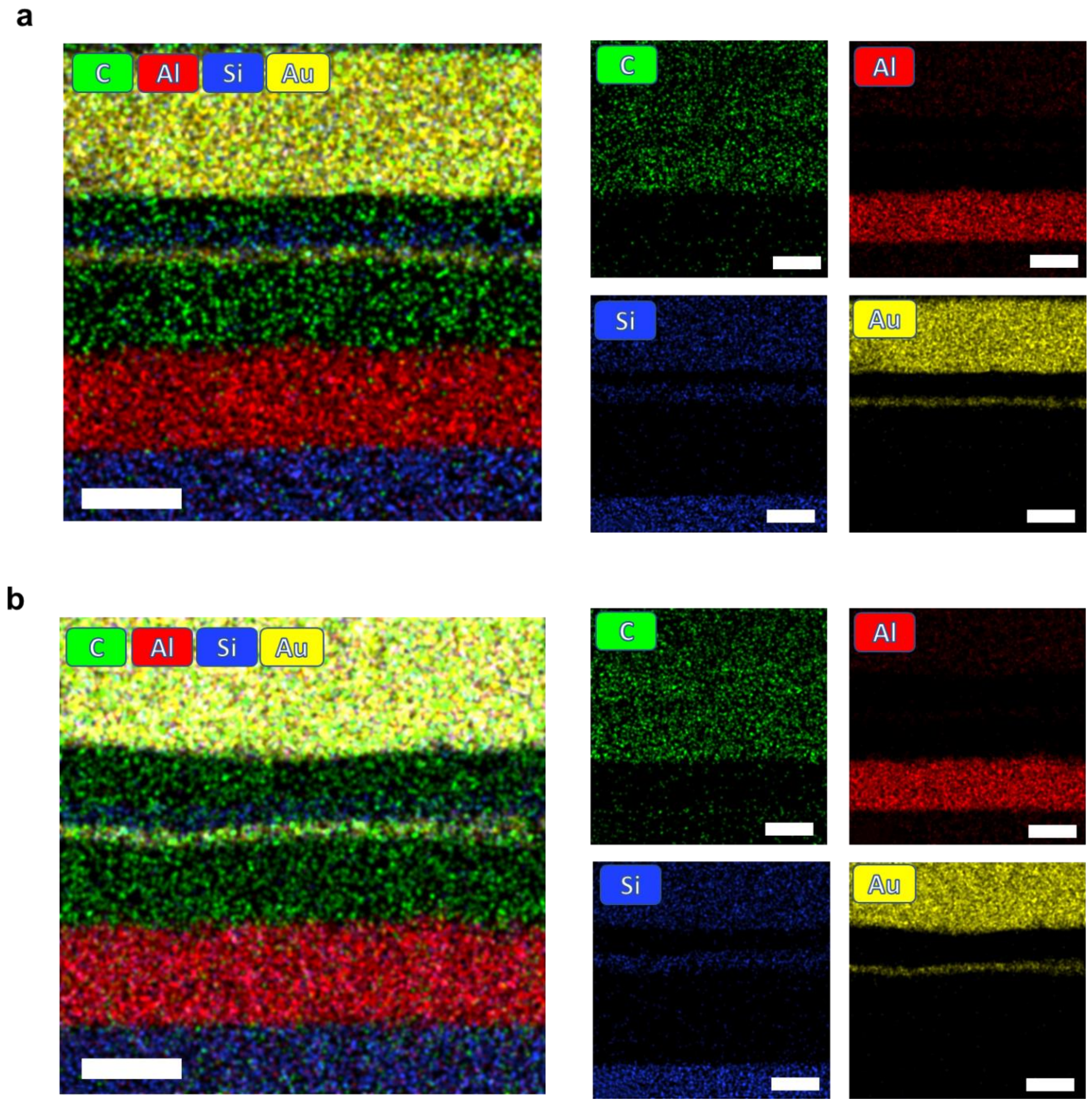

Supplementary Fig. 4: High-resolution transmission electron microscope images

a,b, The cross-sectional high-resolution transmission electron microscope (HRTEM) image and energy dispersive spectroscopy (EDS) elemental mapping result of the n-type (**a**) and p-type part(**b**) of Gaussian-like memory transistor (GMT). (scale bar = 50nm)

The operating principle of the anti-ambipolar transistor

The one electrode formed a contact with a stacked n-type semiconductor and the other electrode contacted a p-type semiconductor only. The heterojunction of PTCDI-C13 and pentacene makes the p-n junction at the channel, and the output current of this anti-ambipolar transistor was affected by the band-to-band tunneling of conduction band of n-type to valance band of p-type[7]. The operation principle can be divided into four different regions, as shown in Supplementary Fig. 5[7]:

First step is off-state (gate voltage (V_G) < on voltage (V_{on})): Current follow the corresponds to the off-state of n-type semiconductor due to completely depleted n-channel. Second step is n-type subthreshold region ($V_{on} < V_G <$ voltage corresponding to the maximum current (V_{max})): where p-type semiconductor is fully conducting and n-type one starts to accumulate electron. Third step is p-type subthreshold region ($V_{max} < V_G <$ off voltage (V_{off})): where n-type region is fully conducting, whereas the amount of charges in the p-type semiconductor is decreased with V_G, as the p-type pentacene is depleted by the positive gate voltage bias. Last step is off-state ($V_{off} < V_G$): due to the p-type pentacene semiconductor completely depleted and become off-state.

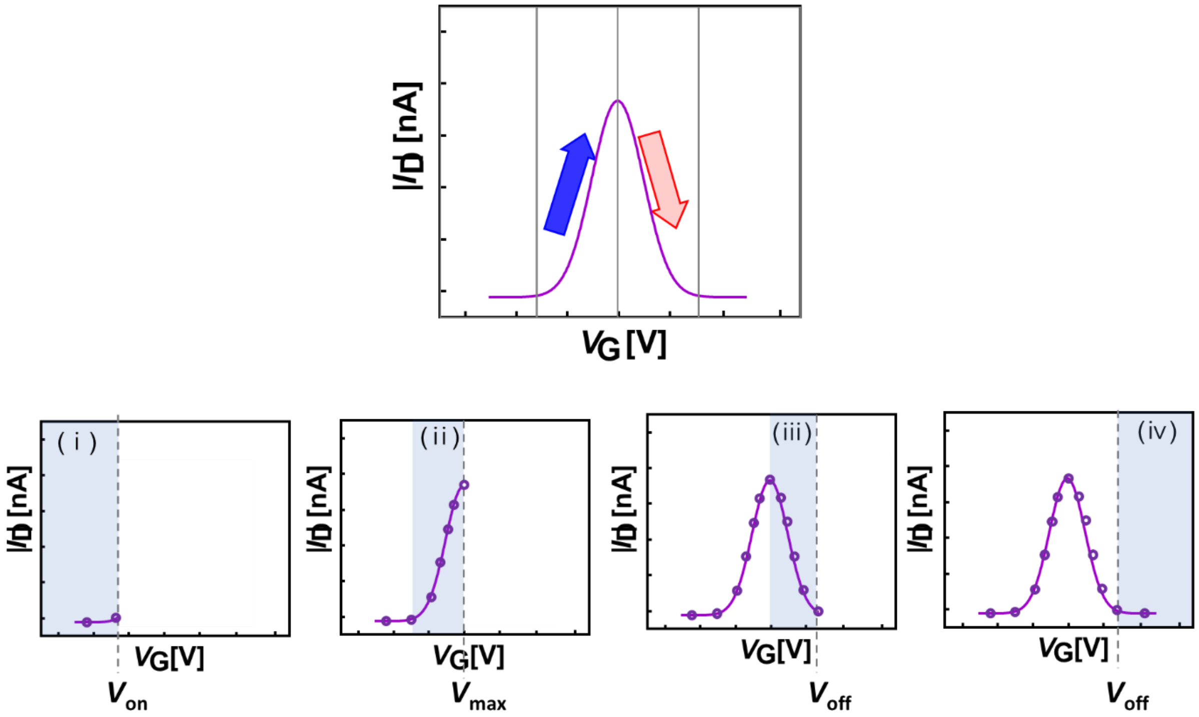

Supplementary Fig. 5: Operating principle of anti-ambipolar transistor

The operating principle of the anti-ambipolar transistor divided by four distinguishable regimes. The shaded area represents the region of each state.

The operating principle of the anti-ambipolar transistor with different V_D

This behavior can be explained by the device structure, where the n-channel is connected to the source (S) terminal that is set to ground (GND) and the p-channel is connected to drain (D) terminal, which experiences the applied V_D. Consequently, V_{on} is dependent on the n-channel accumulation and the applied electric field (E) to the n-channel, which remains unaffected with V_D variation, leading to a constant V_{on}. On the other hand, the applied E on p-channel is influenced by the difference between V_D and V_G, resulting in the variation of V_{off} with the change in V_D[8].

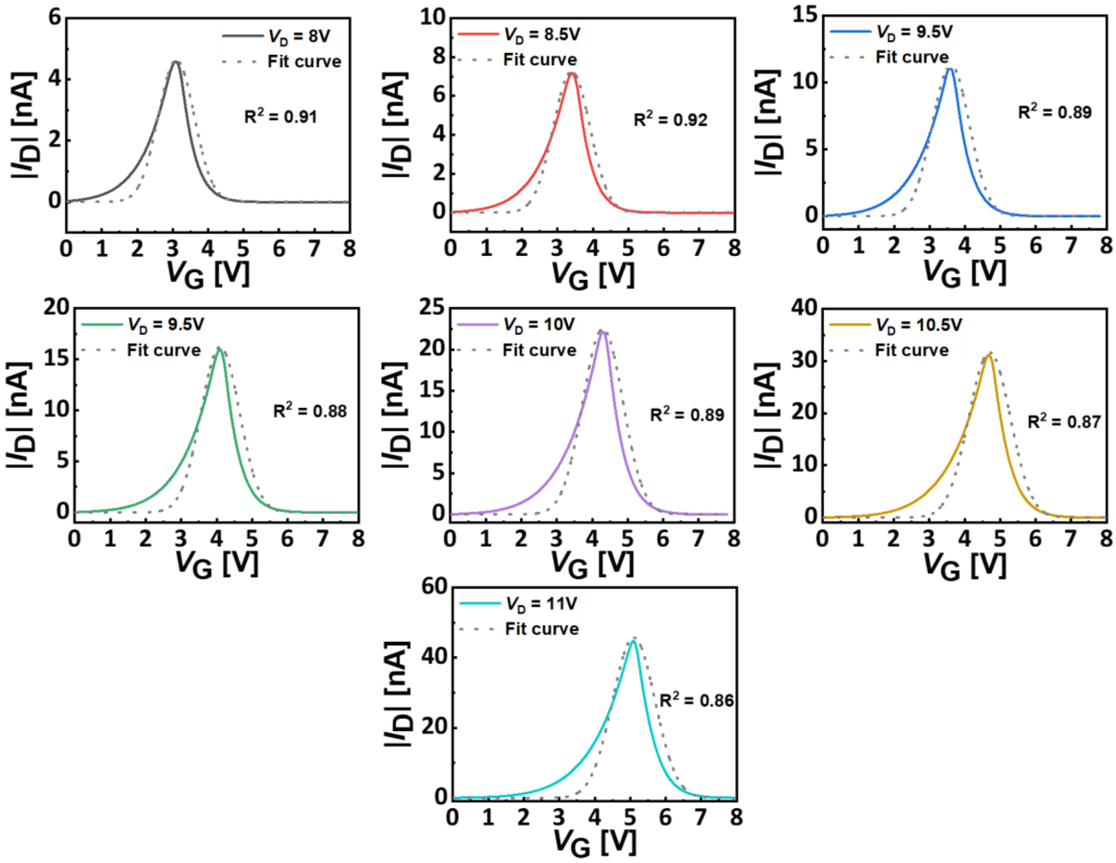

Supplementary Fig. 6: Transfer characteristics with different drain voltage.
Transfer characteristics (absolute value of drain current ($|I_D|$) verse gate voltage (V_G)) with different drain voltage (V_D) 8V to 11V and a result of fitting according to the Gaussian formula.

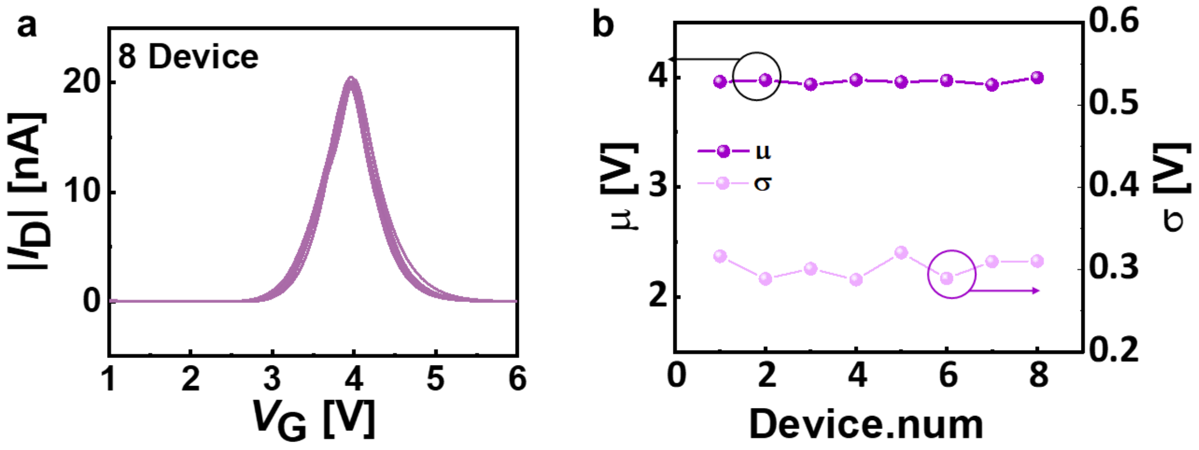

Supplementary Fig. 7: Device uniformity.

a, Transfer characteristics (absolute value of drain current ($|I_D|$) verse gate voltage (V_G)) with 8 different devices. **b,** Fitting result of mean (μ) and standard deviation (σ) values.

Individual programming of GMT device

Supplementary Fig. 8 shows OM (Optical Microscope) and AFM (Atomic Force Microscope) images depicting the fabrication process of the GMT (Gaussian-like Memory Transistor) device. With the positive $V_{\mathrm{prg,N}}$ was applied, accumulated electrons in the n-type semiconductor could pass through the tunneling dielectric layer by Fowler-Nordheim (F-N)-like tunneling and be trapped in the AuNP FG[1, 9]. (Supplementary Fig. 9) The stored electrons induced an additional negative voltage, causing the $V_{\mathrm{T,N}}$ shift toward positive direction. Since charge movement was induced by the electrical potential difference between *S* (or *D*) and gate electrode, electrons could only be stored into the desired FG without affecting the other FG in each programming process. Therefore, only the $V_{\mathrm{T,N}}$ could be shifted toward positive direction without any change in $V_{\mathrm{T,P}}$ (Fig. 3c). As a result, the The maximum I_D ($I_{\max}$) decreased, and $V_{\max}$ was shifted to the right. Conversely, with the applied negative $V_{\mathrm{prg,N}}$, holes can be stored in the n-type FG, which induces negative $V_{\mathrm{T,N}}$ shift, which led to the increased $I_{\max}$ and $V_{\max}$ shift to the left. (Supplementary Fig. 10) Likewise, the positive (negative) $V_{\mathrm{prg,P}}$ induced positive (negative) shift of $V_{\mathrm{T,P}}$, resulting in an increase (decrease) of the $I_{\max}$ and a leftward (rightward) shift of $V_{\max}$. Since charge movement was induced by the electrical potential difference between S (or D) and gate electrode, they electrons (or holes) could only be stored into the desired FG without affecting the other FG in each programming process. Therefore, only the one side of positive direction $V_{T,N}$ could be shifted toward positive direction movement is made without any change in another $V_{T,P}$ change. (Supplementary Figs. 11 b,c). As a result, the maximum I_D decreased, and $V_{\max}$ was shifted to the right. This shift made maximum point move downward to the right along the p-channel conductance. Conversely, with the applied negative $V_{prg,N}$, holes were can be stored in the n-type FG, which creates only induces negative $V_{T,N}$ movement shift, which led to the increased $I_{\max}$ and $V_{\max}$ shift to the left. and the maximum point moves upward to the left along the p-channel conductance. Likewise, The positive

(negative) $V_{prg,P}$ made induced positive (negative) shift of $V_{T,P}$, which makes resulting in an increase (decrease) of the $I_{\max}$ and a leftward (rightward) shift of $V_{\max}$. maximum point moves upward to the right along the n-channel conductance. The negative $V_{T,P}$ shift according to the negative $V_{prg,P}$ caused the maximum point to move downward to the left along the n-channel conductance. (Supplementary Figs. 11 d,e) $I_{\max}$ value and $V_{\max}$ with different programming voltage was shown in Supplementary Fig. 12.

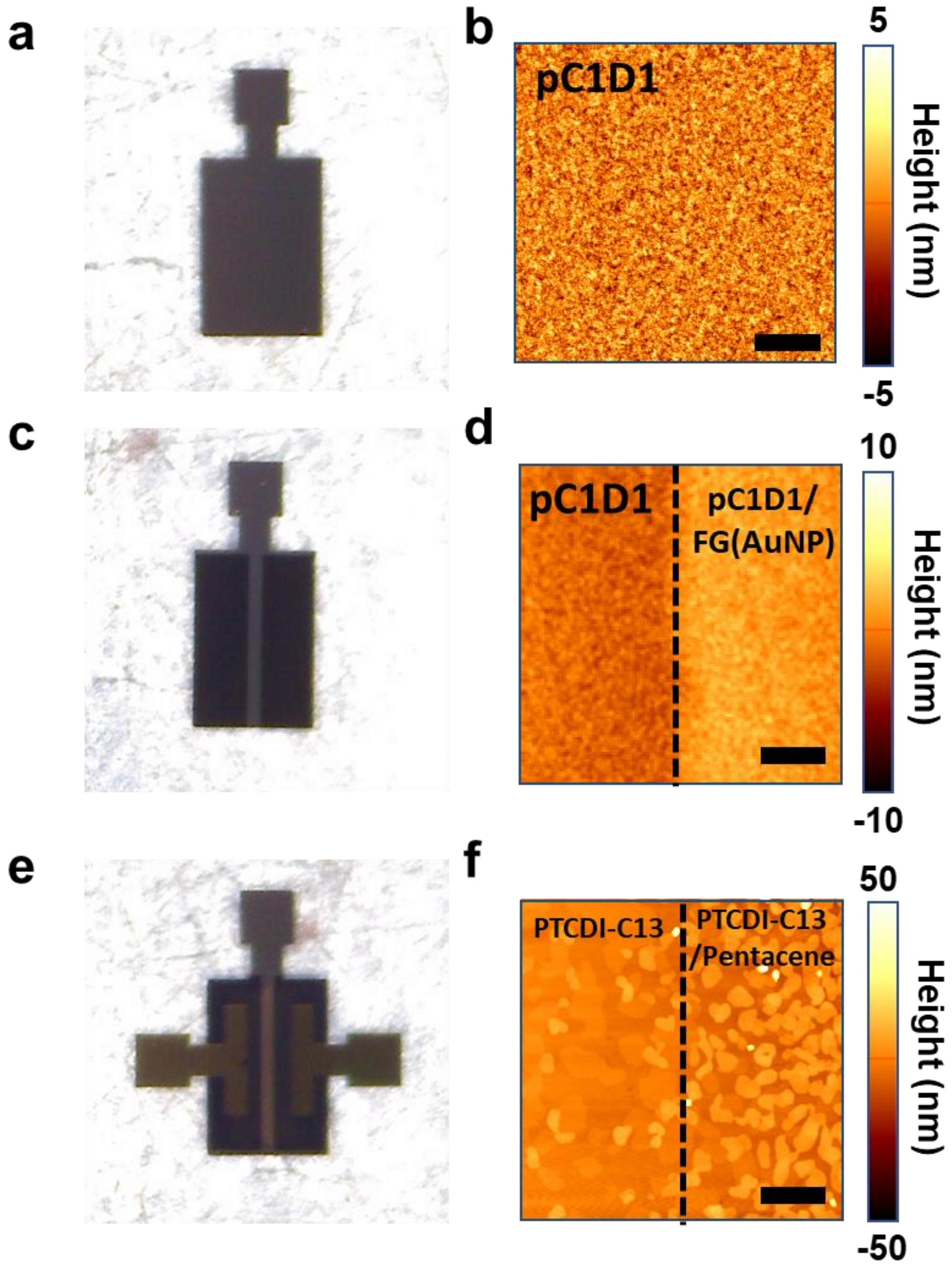

Supplementary Fig. 8: Surface characteristics

a,b, The optical microscope (OM) images (**a**) and atomic force microscope (AFM) images (b) of poly(2-cyanoethyl acrylate-co-diethylene glycol divinyl ether) [p(CEA-co-DEGDVE)] (pC1D1) dielectric. **c,d,** OM images (**c**) and AFM images of gold nanoparticle (AuNP) floating gate (FG) on pC1D1 (**d**), **e,f,** The OM images (**e**) and AFM images (**f**) of middle of the active layer (scale bar = 1um).

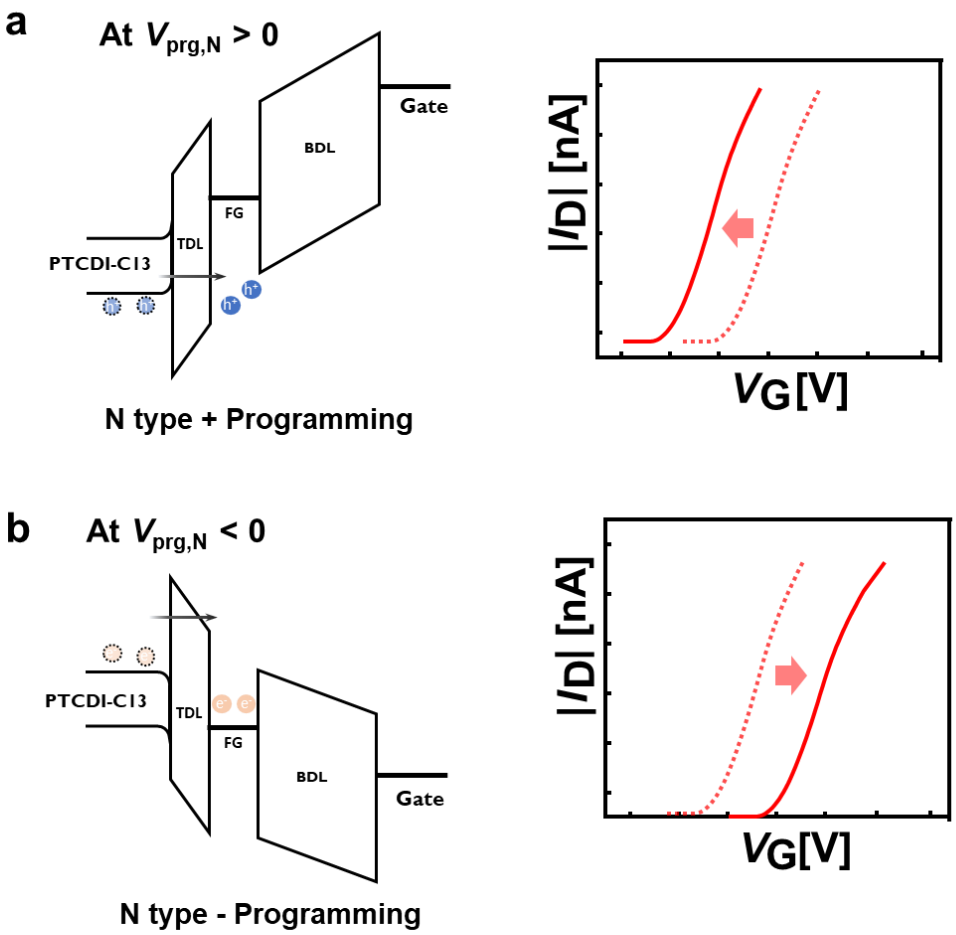

Supplementary Fig. 9: Mechanism of n-type programming

a,b, A band diagram of the Gaussian-like memory transistor (GMT) device n-channel memory operation and corresponding schematic illustration of transfer curve shift along with positive programming (**a**) and negative programming (**b**) operation with the n-type programming voltage ($V_{prg,N}$).

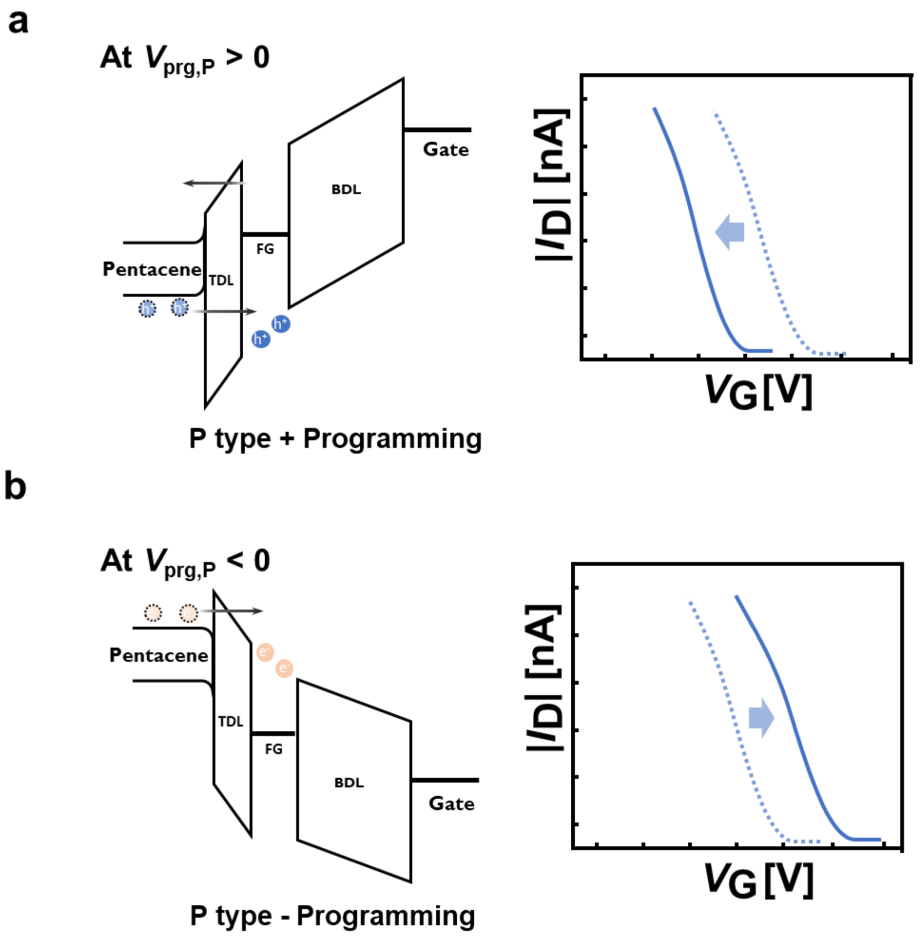

Supplementary Fig. 10: Mechanism of p-type programming

a,b, A band diagram of the Gaussian-like memory transistor (GMT) device p-channel memory operation and corresponding schematic illustration of transfer curve shift along with positive programming (**a**) and negative programming (**b**) operation with the n-type programming voltage ($V_{\mathrm{prg,P}}$).

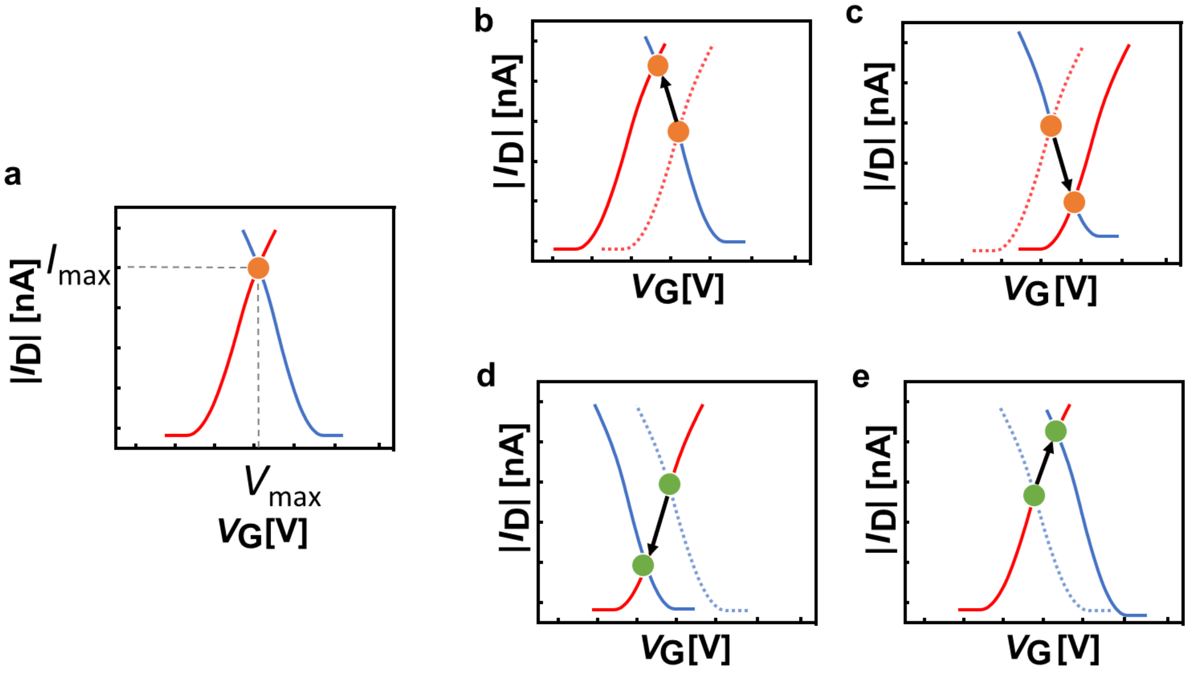

Supplementary Fig. 11: Schematic illustration of movement of transfer curve.

a,b,c,d,e, The schematic illustration Gaussian-like memory transistor (GMT) device transfer curve showing V_G corresponding to the maximum I_D value (V_{max}) (**a**) and the change along with independent programming condition with the negative threshold voltage of n-channel ($V_{T,N}$) movement (**b**), positive $V_{T,N}$ movement (c), negative threshold voltage of n-channel ($V_{T,P}$) movement (**d**), and positive $V_{T,P}$ movement (**e**).

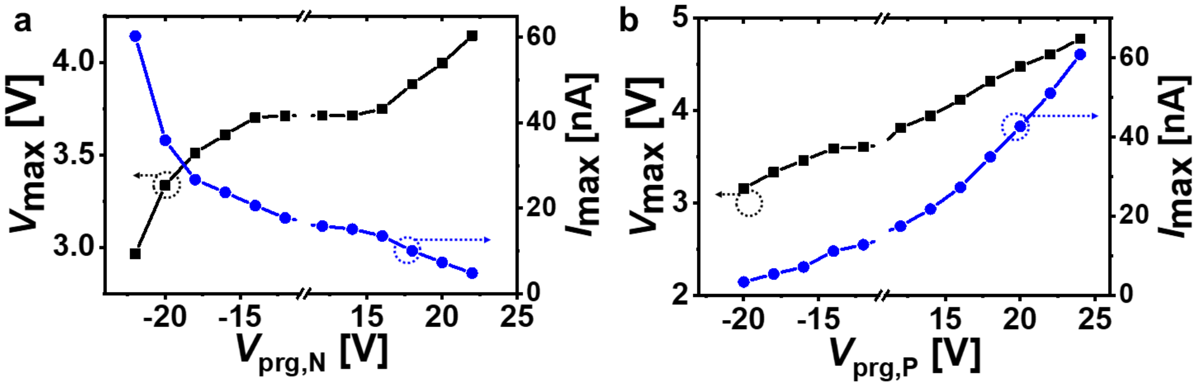

Supplementary Fig. 12: Maximum point movement.

a,b, The difference between maximum I_D (I_{max}) value and V_G corresponding to the I_{max} value maximum point gate voltage (V_{max}) with different programming voltage of n-type (**a**) and p-type (**b**) independent programming condition.

a

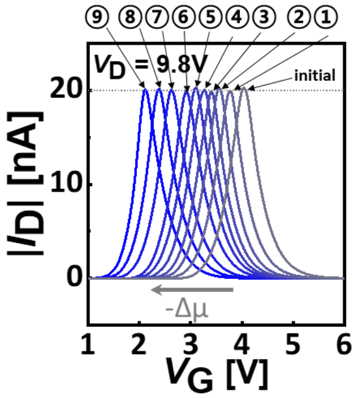

	P	N
1	-17V	-16V
2	-18V	-17V
3	-19V	-18V
4	-20V	-19V
5	-21V	-20V
6	-22V	-21V
7	-23V	-22V
8	-24V	-23V
9	-25V	-24V

b

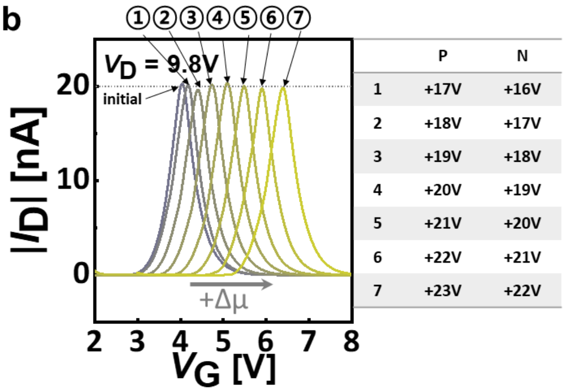

	P	N
1	+17V	+16V
2	+18V	+17V
3	+19V	+18V
4	+20V	+19V
5	+21V	+20V
6	+22V	+21V
7	+23V	+22V

c

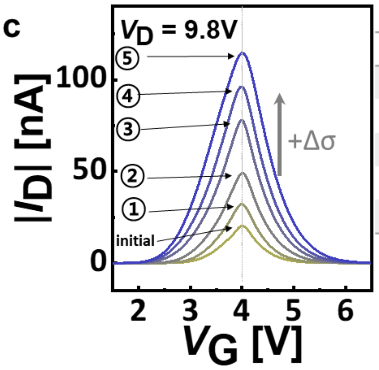

	P	N
1	+16V	-15V
2	+17V	-17.5V
3	+19.5V	-19V
4	+20.5V	-20V
5	+21.5V	-21V

d

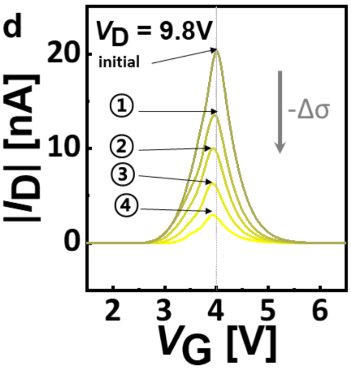

v	P	N
1	-18.V	+18.5V
2	-19.5V	+19.5V
3	-21.5V	+20V
4	-22V	+20.5V

Supplementary Fig. 13: Programming condition.

a,b, Programming condition corresponding change in mean (μ) value with respect to negative direction (**a**), and positive direction (**b**). **c,d,** Programming condition corresponding change in standard deviation (σ) value with respect to positive direction (**c**), and negative direction (**d**).

Factors of Gaussian distribution and three sigma rules

In statistics, the 68-95-99.7 rule, also known as the empirical rule, is a guideline that applies to a normal distribution. (Supplementary Fig. 14) It is sometimes referred to as the three-sigma rule, indicating that nearly all values (99.7%) fall within a range of three standard deviations from the mean in both directions[10].

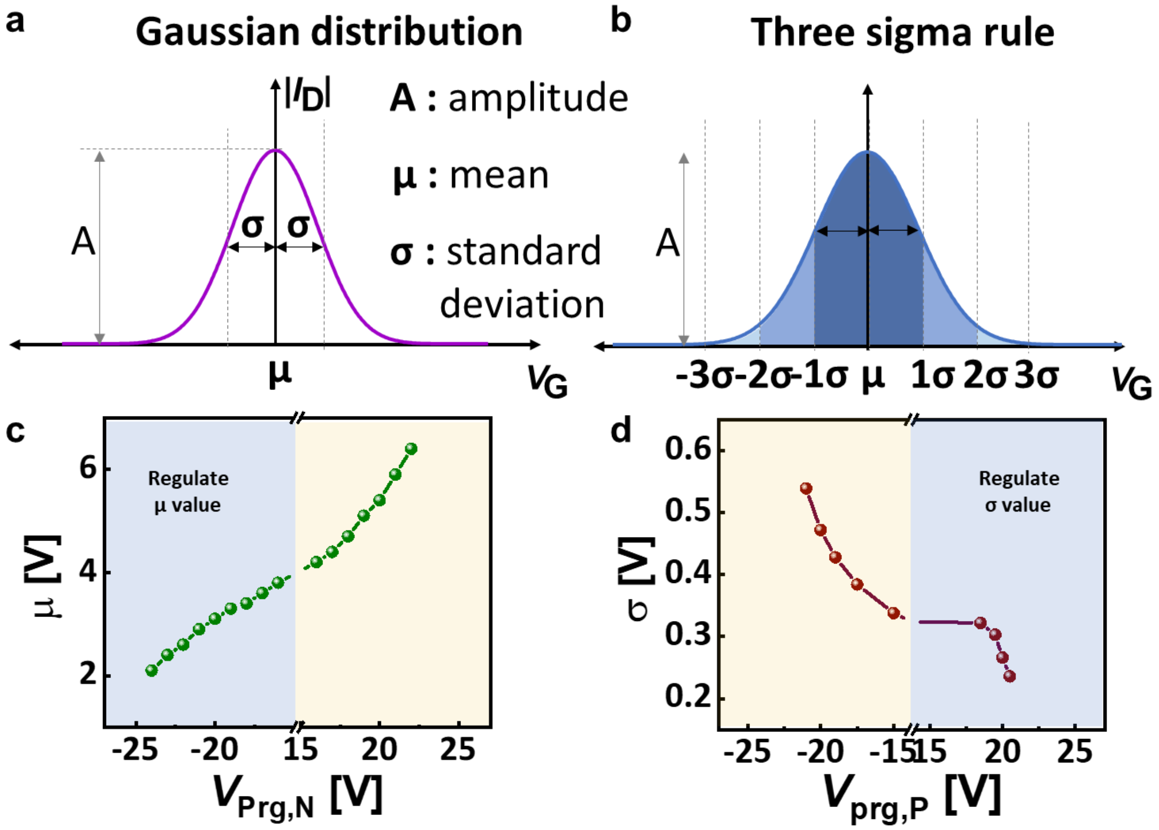

Supplementary Fig. 14: Three sigma rules.

a, A schematic illustration Gaussian-like distribution shape and the three factors (amplitude (A), mean (μ), standard deviation (σ). **b,** A schematic illustration of three sigma rule. **c,** Corresponding change in μ value with respect to n-type programming voltage ($V_{prg,N}$) with the programming of the change of the threshold voltage of p-channel ($\Delta V_{T,P}$) = the change of the threshold voltage of p-channel ($\Delta V_{T,N}$) . **d,** Corresponding change in σ value with respect to $V_{prg,N}$ with the programming of $\Delta V_{T,P} = - \Delta V_{T,N}$.

Stability and low power operation of GMT device.

The retention characteristics of the fabricated GMT device exhibited stability in both mean and standard deviation, when extrapolated up to 10^8 s. (Supplementary Fig. 15) Additionally, optimization of the insulator thickness confirmed the potential for reducing operating voltage. The device utilizing 70 nm p(CEA-co-DEGDVE) blocking layer and 8 nm pV3D3 tunneling layer. Using the GMT device with the reduced insulating layer thicknesses, with V_D = 4.5 V, we observed output current values. Furthermore, when programming the fabricated devices, we successfully achieved the systematic movement of a Gaussian-like-shaped graph without device failure. (Supplementary Fig. 17)

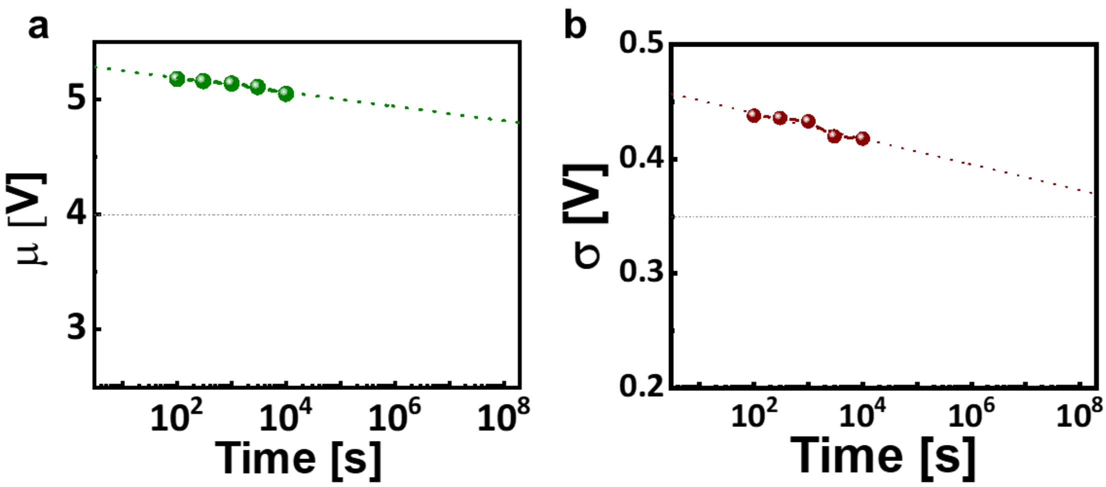

Supplementary Fig. 15: Extrapolation of retention characteristics
a,b, The retention characteristics of Gaussian-like memory transistor (GMT) device in applied time versus mean (μ) (**a**) and standard deviation (σ) (**b**) value change which were extrapolated to 10 years.

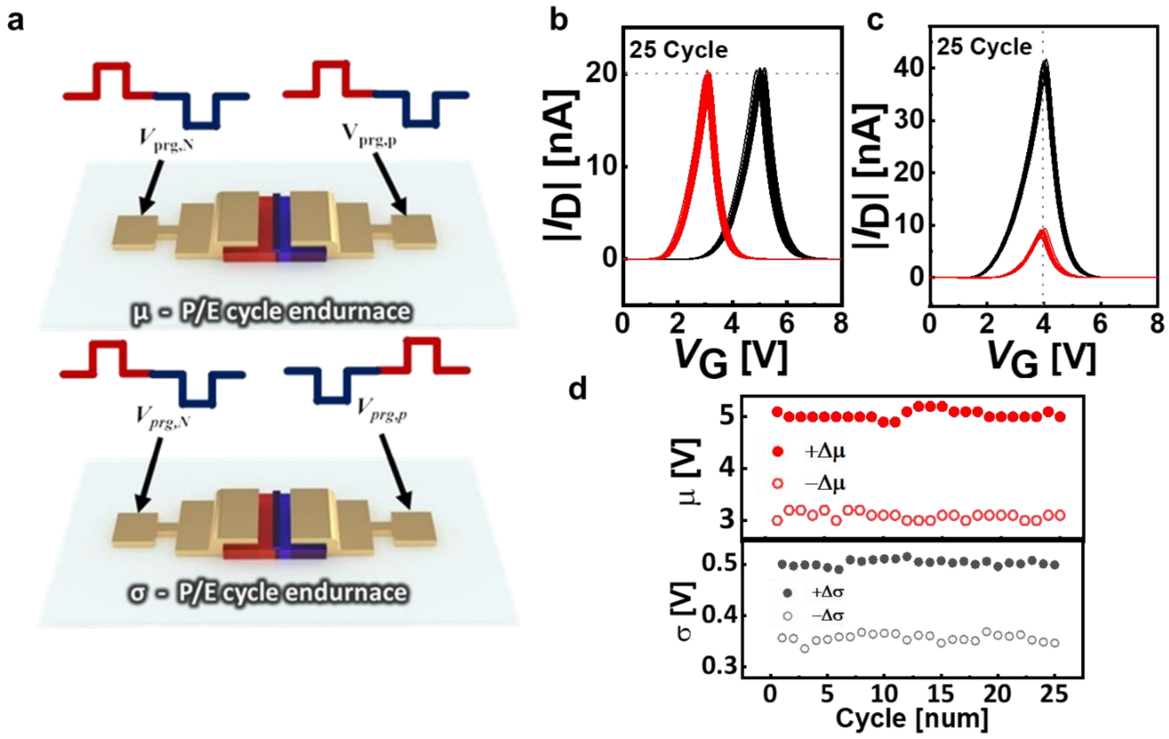

Supplementary Fig. 16: Programming and erasing cycle endurance test.

a, A schematic diagram of cycle endurance measurement of Gaussian-like memory transistor (GMT) device. **b**,**c,** The transfer curves of repetitive programming and erasing operations up to 25 cycles with mean (μ) (**b**) and standard deviation (σ) regulation (**c**). **d,** The change in μ value(red) and σ value (black) according to the endurance cycle.

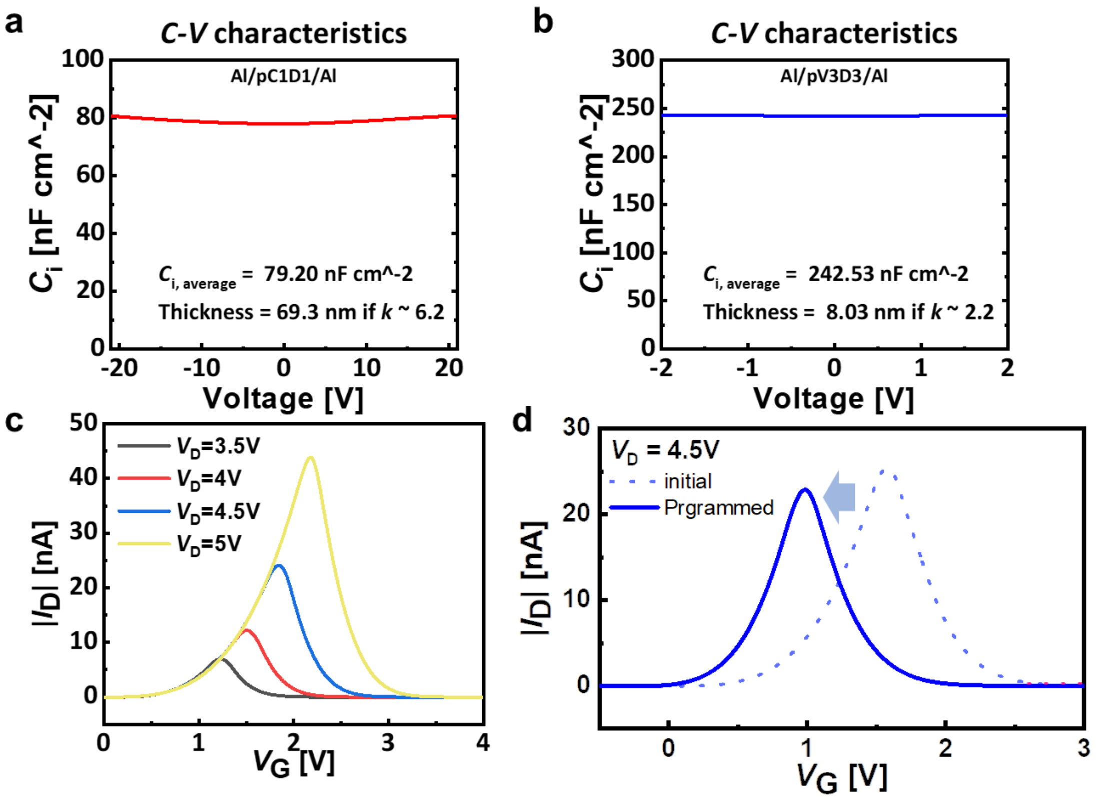

Supplementary Fig. 17. The electrical characteristics of fabricated GMT device.
a,b, The capacitance (C)–voltage (V) characteristics of blocking dielectric layer (BDL)(Al/ poly(2-cyanoethyl acrylate-co-diethylene glycol divinyl ether) [p(CEA-co-DEGDVE)] (pC1D1)/Al metal-insulator-metal (MIM) device) (**a**) and tunneling dielectric layer (TDL) (Al/ poly(1,3,5-trivinyl-1,3,5-trimethyl cyclotrisiloxane) (pV3D3) /Al MIM) (**b**). **c,** The transfer characteristics fabricated GMT device. **d,** The transfer characteristics measured in parallel movement programming (mean (μ) value change).

Simulation for insect size-drones mapping model with GMT device data

Recent advancements in robotics have led to the development of miniature designs weighing just a few grams and measuring a few centimeters. Notable examples include, RoboFly, an insect-scale robot with a weight of only 100 mg and a wingspan of 30 mm[11] and RoboBees, weighing a mere 80 mg with rotor dimensions of just 30 mm[12]. Equipping these tiny robots with autonomous navigation capabilities further opens up a world of intriguing possibilities. For instance, an autonomous insect-scale drone can navigate through narrow passages, maneuver in cluttered environments, and collaborate in swarms, enabling it to tackle tasks that would be challenging or even impossible for larger drones. However, due to their limited carrying capacity, insect-scale drones and robots can only accommodate a small battery for power. Therefore, minimizing power consumption and footprint for onboard processing for autonomous navigation becomes crucial. We anticipate that by disruptively enhancing energy and area-efficiency of probabilistic reasoning computations, GMTs can become a leading technology to open these application pathways.

Localization is a crucial operation in drone and robot autonomy, wherein a vehicle determines its position and orientation by utilizing sequential measurements. Localization is commonly achieved using particle filtering, a Monte Carlo technique that implements sequential belief updates based on measurements[13]. This approach involves considering a set of hypotheses (particles), where each particle is assigned a weight index that is updated using sequential measurements. Through successive calculations, particles with low likelihoods are filtered out, and a new set of particles is generated to account for uncertainties in motion control. By leveraging Bayes' rule, this framework seamlessly integrates single-shot maximum a posteriori (MAP) estimates of robot positions with sequential localization stages.

While particle filtering can handle arbitrary uncertainty distributions, it is computationally demanding. One of the main complexities arises from estimating the likelihood term due to the

high dimensionality of measurements, flying domain complexity, and considered hypotheses. Typically, the flying domain is modeled using a Gaussian Mixture Model (GMM) based on point-cloud data obtained from scanning devices. The parameters of the GMM, including the number of mixture components, are learned using the Expectation-Maximization procedure[14]. Subsequently, depth measurements under a test hypothesis are projected onto the GMM-modeled domain map, as shown in Fig. 6d, and the likelihood of the measurements under the hypothesis is computed. If the measurements are sufficiently likely under the hypothesis, they are retained for subsequent estimations; otherwise, they are discarded. The likelihood computations are expensive as they need to be iterated over all considered hypotheses and measurement dimensions, while the GMM-represented domain map itself may be complex, i.e., involves many mixture functions, depending on the size and complexity of the flying environment.

We have carefully conceived the envisioned Gaussian-like memory transistor (GMT)-based platform for implementation in the 45 nm channel length technology. This choice is grounded in the fact that commercial products utilizing a comparable floating gate structure, such as NAND/NOR Flash memories, are already present at the 45 nm channel length. Accordingly, power dissipation was estimated using SPICE simulations, relying on predictive technology models referenced in FreePDK^{45TM} 45nm (https://eda.ncsu.edu/freepdk/freepdk45/). Additionally, for a fair benchmark against digital design, we referred to advanced studies. Khan *et al*. demonstrated high-k metal/gate stack programmability at the 14 nm node[15], and industry research presented NAND flash technology scaled to less than the 14 nm node[16]. Therefore, we conducted a digital design of state-of-the-art complementary metal–oxide–semiconductor (CMOS) processes at both 14 nm and 7 nm. The GMT device exhibited slightly enhanced performance compared to digital CMOS devices under 14 nm, as illustrated in Supplementary Table3.

In Supplementary Fig. 18 a, GMT characteristics are mirrored using series-connected NMOS and PMOS, where their gate control is managed through capacitors mimicking GMT's floating gates. Supplementary Fig. 18 b showcases the CMOS circuit equivalent, displaying a Gaussian current output curve where the peak voltage adjustment is feasible by altering floating gate charges while ensuring the peak current remains consistent. Significantly, GMT consolidates these features into one device, maintaining non-volatility and allowing programmability of both mean and sigma, unlike CMOS circuit of Supplementary Fig. 18 a. Thus, for large scale architectures, GMT remains irreplaceable. The CMOS-emulated circuit is presented here merely to underline the energy advantages anticipated with GMT's nanometer node scaling. The data-path for computing likelihood using the emulated GMT architecture is depicted in Fig. 6a. Initially, a digitally represented vector, whose likelihood is under assessment, transitions to the analog domain using digital-to-analog converters (DAC) within the data-path. These analog values undergo voltage amplification to operate against the floating gate of GMT and input capacitors in the emulator circuit in Supplementary Fig. 18 a. Supplementary Fig. 18 c shows the response of voltage gain circuit, resulting in <50 ns settling time at various input voltages. In Fig. 6a, depending on the applied voltage and the programmed capacitor voltage (representing the emulated floating gate), cells across each row generate a current indicative of the input vector's likelihood. This emergent cell current undergoes digitization in the log-domain using a log-ADC. Subsequently, the log-ADC output undergoes multiplication, computing the likelihood score as elaborated in Eq. (3), in accordance with the mean field approximation. Fig. 6c presented a comparative digital data-path for evaluating the likelihood. In contrast to GMT-based designs, the digital data-path's parallelism is inherently restricted due to the presence of large computational modules. Specifically, modules like the multiply-accumulate (MAC) unit and the squaring circuit (x^2) require hundreds of transistors. Consequently, given the constraints on area and power, only a limited number of parallel digital

data-paths can be feasibly implemented. Each data-path core can also process the input data only sequentially. For every element of the feature vector, the deviation is computed by contrasting it with the stored mean vector. This difference is then squared using the x^2 circuit. The outputs from all the feature vector elements are aggregated using the MAC circuit. Following this, a log-ADC circuit calculates the logarithmic likelihood. This is achieved by referencing a lookup table (LUT) that retains the output values of the log(1+exp(x)) function. Using LUT's iterative evaluation, as discussed in previous research[17], the net likelihood is computed in the digital domain. By comparing the GMT-based architecture against digital data-path, critical advantages of GMT are evident. By leveraging the characteristics of GMT, the number of GMT devices are only as many as in the product of the dimension of the likelihood function and the total number of mixture functions. Since each mixture function can be implemented in a single GMT device, typical drone localization and planning operations can be mapped onto a network of 100 – 300 GMTs. Moreover, unlike the CMOS design, each GMT operates in parallel to significantly outperform sequential processing of digital accelerators. Although our implementation requires log-ADC and DAC to operate in analog domain, overheads of these circuits amortize on a larger processing array operating on complex likelihood functions.

In Supplementary Fig. 19 a, consider a series connection of GMTs. Here, gate-controlled conductance of each GMT follows a Gaussian-like behavior as described in Eq. (2). At lower drain biasing voltages (V_{DS}), GMTs operate under diffusion and linearity of their current against V_{DS} improves as shown in Supplementary Fig. 19 b. Therefore, for a series connection of GMTs, under lower column biasing voltage, the column current follows a *Harmonic mean of their Gaussian-like* (HMGL) characteristics as shown in Supplementary Figs. 19 a, c and d show the surface and contour profile of the resultant HMGL function emulated by the column current of series-connected GMTs. Notably, similar to a Gaussian function, HMGL is a single

mode function. However, unlike a Gaussian function, it follows rectilinear surface tails as shown in Supplementary Fig. 19 d.

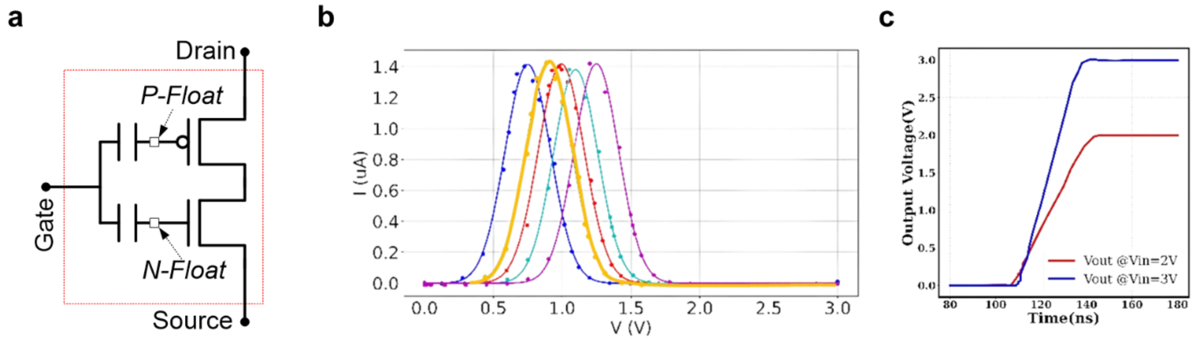

Supplementary Fig. 18. complementary metal–oxide–semiconductor emulation.
a, complementary metal–oxide–semiconductor (CMOS) emulation of Gaussian-like Memory Transistor (GMT). **b,c,** Simulated characteristics of (**b**) CMOS emulation using 45 nm predictive technology models and (**c**) input voltage application stage to emulated GMT array.

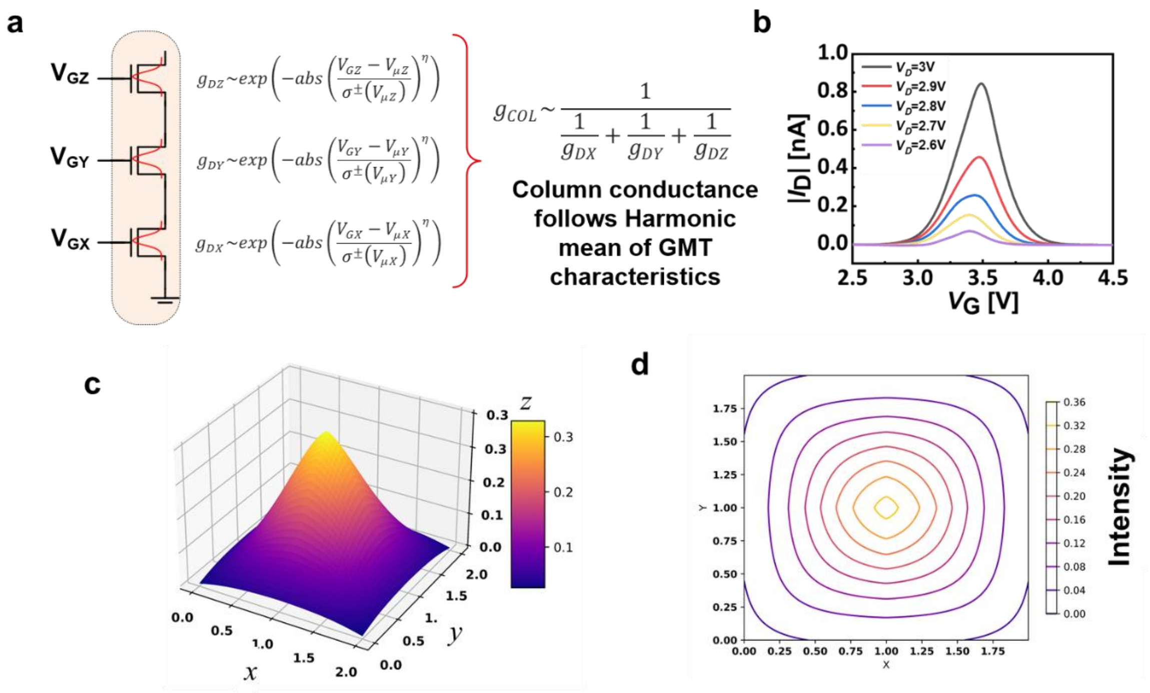

Supplementary Fig. 19. 3-dimensional emulation.

a, Series connection of Gaussian-like memory transistors (GMTs), while biasing the column at low voltages, induces a Harmonic mean of GMT characteristics. **b,** The transfer curves of low drain voltage (V_D) 2.6V to 3V with the subthreshold operation. **c,d,** Surface plot (**c**) and contour plot (**d**) of normalized column current of three series-connected GMTs. The current profile shows a single mode peak and rectilinear surface tails.

Supplementary Table 1. 8-bit Digital Datapath's Energy for Likelihood Computation using 100-Mixture, 3-Dimensional Gaussian Mixture Model (GMM) in CMOS 14 nm & CMOS 7 nm.

**Operation Type**	**# of Operations**	**Energy/Op. (fJ) (CMOS 7 nm)**	**Energy/Op. (fJ) (CMOS 14 nm)**	**Energy/Op. (fJ) (CMOS 45 nm)**
ADD/SUB	10699	0.325[18]	0.72[18]	30[19]
Multiplication	600	1.004[18]	3.307[18]	200[19]
Read	100	230[20]	920[20]	5000[21]
**Total Energy**		**27.08 pJ**	**101.68 pJ**	**941 pJ**

Supplementary Table 2. 8-bit GMT-based Mixed-Signal Datapath's Energy for Likelihood Computation using 100-Mixture, 3-Dimensional Density Function in Eq. 3 at 45 nm.

Operation	Energy/Operation (pJ)[22, 23]	# of Operations	Energy (pJ)
8-bit DAC	0.94[22]	3	2.82
Voltage Gain	1.98	3	5.94
GMT Array Dynamic	2.15	1	2.15
GMT Array Bias	5.04	1	5.04
8-bit log-ADC	2.38[23]	1	2.38
**Total Energy**			**18.33J**

Supplementary Table 3. Compare of energy consumption of GMT and CMOS devices

	GMT (45 nm)	Digital CMOS (14 nm)	Digital CMOS (7 nm)
Energy/ Likelihood Step	18.33 pJ	101.7 pJ	27.1 pJ
Improvement		5.5×	1.48×

Supplementary Table 4. Summary of the electrical characteristics of the reported anti-ambipolar transistors

Year[ref]	Operating voltage	Semiconductor	Dielectric
2016[24]	40	MoS_2/WSe_2	SiO_2
2018[25]	80	MoS_2/tetracene	SiO_2
2018[26]	10	α-6T/PTCDI-C8	PMMA/Al_2O_3
2019[7]	50	PTCDI-C13/DNTT	SiO_2
2019[27]	20	MoS_2/BP	SiO_2
2020[28]	60	H-Type/TTT-CN	SiO_2
2020[29]	6	MoS_2/CNT	Al_2O_3
2020[30]	10	- (simulation)	PMMA/Al_2O_3
2021[31]	60	PTCDI-C13/DNTT	SiO_2
2021[32]	50	PTCDI-C13/DNTT	-
2021[33]	30	InSe/WSe_2	hBN
2021[34]	80	inSe/2H-$MoTe_2$	SiO_2
2022[35]	15	WSe_2/ReS_2	SiO_2
2022[36]	40-100	DBTTF/cyh-NDI	SiO_2
**This work**	**10**	PTCDI-C13/pentacene	**iCVD dielectric**

Supplementary Table 5. Summary of the performance of reported Gaussian like *I-V* characteristics system

Ref num	Number of Transistor	Input variable	Regulate factor
1975[37]	4	3	μ
1993[38]	7	3	μ
2013[39]	22	5	μ,σ,A
2014[40]	31	8	μ,σ,A
2019[41]	15	4	μ,σ,A
2021[42]	14	5	μ,σ,A
2022[43]	10	3	$\boldsymbol{\mu,\sigma,A}$
2022[44]	18	4	$\boldsymbol{\mu,\sigma,A}$
**This work**	**1**	**1**	$\boldsymbol{\mu,\sigma,A}$

Supplementary References

1. Yang SC, *et al.* Large‐scale, low‐power nonvolatile memory based on few‐layer MoS2 and ultrathin polymer dielectrics. *Adv Electron Mater* **5**, 1800688 (2019).

2. Moon H, *et al.* Synthesis of ultrathin polymer insulating layers by initiated chemical vapour deposition for low-power soft electronics. *Nat Mater* **14**, 628-635 (2015).

3. Lee S, Seong H, Im SG, Moon H, Yoo S. Organic flash memory on various flexible substrates for foldable and disposable electronics. *Nat Commun* **8**, 725 (2017).

4. Lee C, Pak K, Choi J, Kim MJ, Cho BJ, Im SG. Long‐term retention of low‐power, nonvolatile organic transistor memory based on ultrathin, trilayered dielectric containing charge trapping functionality. *Adv Funct Mater* **30**, 2004665 (2020).

5. Choi J, *et al.* Vertically stacked, low-voltage organic ternary logic circuits including nonvolatile floating-gate memory transistors. *Nat Commun* **13**, 2305 (2022).

6. Choi J, *et al.* Flexible, low-power thin-film transistors made of vapor-phase synthesized high-k, ultrathin polymer gate dielectrics. *ACS Appl Mater Interfaces* **9**, 20808-20817 (2017).

7. Yoo H, On S, Lee SB, Cho K, Kim JJ. Negative transconductance heterojunction organic transistors and their application to full‐swing ternary circuits. *Adv Mater* **31**, 1808265 (2019).

8. Hayakawa R, Honma K, Nakaharai S, Kanai K, Wakayama Y. Electrically Reconfigurable Organic Logic Gates: A Promising Perspective on a Dual‐Gate Antiambipolar Transistor. *Adv Mater* **34**, 2109491 (2022).

9. Woo MH, *et al.* Low‐power nonvolatile charge storage memory based on MoS2 and an ultrathin polymer tunneling dielectric. *Adv Funct Mater* **27**, 1703545 (2017).

10. Pukelsheim F. The three sigma rule. *The American Statistician* **48**, 88-91 (1994).

11. Chukewad YM, James J, Singh A, Fuller S. RoboFly: An insect-sized robot with simplified fabrication that is capable of flight, ground, and water surface locomotion. *IEEE Transactions on Robotics* **37**, 2025-2040 (2021).

12. Mange S, Helbling EF, Gravish N, Wood RJ. An actuated gaze stabilization platform for a flapping-wing microrobot. In: *2017 IEEE International Conference on Robotics and Automation (ICRA)*). IEEE (2017).

13. WALLÉN KIESSLING A. Monte Carlo Localization with Hilbert maps as Likelihood Fields.) (2023).

14. Reddy CK, Chiang H-D, Rajaratnam B. Trust-tech-based expectation maximization for learning finite mixture models. *IEEE Transactions on Pattern Analysis and Machine Intelligence* **30**, 1146-1157 (2008).

15. Khan F, Cartier E, Woo JC, Iyer SS. Charge trap transistor (CTT): An embedded fully logic-compatible multiple-time programmable non-volatile memory element for high-$ k $-metal-gate CMOS technologies. *IEEE Electron Device Lett* **38**, 44-47 (2016).

16. Yoon C-W. The Fundamentals of NAND Flash Memory: Technology for tomorrow's fourth industrial revolution. *IEEE Solid-State Circuits Magazine* **14**, 56-65 (2022).

17. Price M, Glass J, Chandrakasan AP. A 6 mW, 5,000-word real-time speech recognizer using WFST models. *IEEE Journal of Solid-State Circuits* **50**, 102-112 (2014).

18. Xie Q, Lin X, Wang Y, Chen S, Dousti MJ, Pedram M. Performance comparisons between 7-nm FinFET and conventional bulk CMOS standard cell libraries. *IEEE Transactions on Circuits and Systems II: Express Briefs* **62**, 761-765 (2015).

19. Park S-S, Chung K-S. CENNA: cost-effective neural network accelerator. *Electronics* **9**, 134 (2020).

20. Vashishtha V, Vangala M, Sharma P, Clark LT. Robust 7-nm SRAM design on a predictive PDK. In: *2017 IEEE International Symposium on Circuits and Systems (ISCAS)*). IEEE (2017).

21. Pal B, Chatterjee K. Design of Low Power 14T SRAM using 45 nm CMOS Technology. *International Journal of Engineering Research & Technology (IJERT)* **8**, 1-6 (2019).

22. Hong H-K, *et al.* A decision-error-tolerant 45 nm CMOS 7b 1 GS/s nonbinary 2b/cycle SAR ADC. *IEEE Journal of Solid-State Circuits* **50**, 543-555 (2014).

23. Lee J, *et al.* A 2.5 mw 80 db dr 36 db sndr 22 ms/s logarithmic pipeline adc. *IEEE Journal Of Solid-State Circuits* **44**, 2755-2765 (2009).

24. Li Y, *et al.* Anti-ambipolar field-effect transistors based on few-layer 2D transition metal dichalcogenides. *ACS Appl Mater Interfaces* **8**, 15574-15581 (2016).

25. Park HJ, Park C-J, Kim JY, Kim MS, Kim J, Joo J. Hybrid characteristics of MoS2 monolayer with organic semiconducting tetracene and application to anti-ambipolar field effect transistor. *ACS Appl Mater Interfaces* **10**, 32556-32566 (2018).

26. Kobashi K, Hayakawa R, Chikyow T, Wakayama Y. Multi-valued logic circuits based on organic anti-ambipolar transistors. *Nano Lett* **18**, 4355-4359 (2018).

27. Sebastian A, Pannone A, Subbulakshmi Radhakrishnan S, Das S. Gaussian synapses for probabilistic neural networks. *Nat Commun* **10**, 4199 (2019).

28. Liu J, *et al.* High-performance n-and p-type organic single-crystal field-effect transistors with an air-gap dielectric towards anti-ambipolar transport. *J Mater Chem C* **8**, 4303-4308 (2020).

29. Beck ME, *et al.* Spiking neurons from tunable Gaussian heterojunction transistors. *Nat Commun* **11**, 1565 (2020).

30. Kim CH, Hayakawa R, Wakayama Y. Fundamentals of organic anti - ambipolar ternary inverters. *Adv Electron Mater* **6**, 1901200 (2020).

31. On S, Kim Y-J, Lee H-K, Yoo H. Ambipolar and anti-ambipolar thin-film transistors from edge-on small-molecule heterostructures. *Appl Surf Sci* **542**, 148616 (2021).

32. Yoo H, Kim C-H. Unified compact model for thin-film heterojunction anti-ambipolar transistors. *IEEE Electron Device Lett* **42**, 1323-1326 (2021).

33. Paul Inbaraj CR, *et al.* A bi-anti-ambipolar field effect transistor. *ACS nano* **15**, 8686-8693 (2021).

34. Sun Y, *et al.* Anti-ambipolar behavior and photovoltaic effect in p-MoTe 2/n-InSe heterojunctions. *J Mater Chem C* **9**, 10372-10380 (2021).

35. Shingaya Y, *et al.* Dual - Gate Anti - Ambipolar Transistor with Van der Waals ReS2/WSe2 Heterojunction for Reconfigurable Logic Operations. *Adv Electron Mater* **9**, 2200704 (2023).

36. Zhu J, Mori T. Output and Negative - Region Characteristics in Organic Anti - Ambipolar Transistors. *Adv Electron Mater* **9**, 2200783 (2023).

37. Gilbert B. Translinear circuits: A proposed classification. *Electronics letters* **1**, 14-16 (1975).

38. Delbrueck T, Mead C. Bump circuits. In: *Proceedings of International Joint Conference on Neural Networks*) (1993).

39. Vrtaric D, Ceperic V, Baric A. Area-efficient differential Gaussian circuit for dedicated hardware implementations of Gaussian function based machine learning algorithms. *Neurocomputing* **118**, 329-333 (2013).

40. Li F, Chang C-H, Basu A, Siek L. A 0.7V low-power fully programmable Gaussian function generator for brain-inspired Gaussian correlation associative memory. *Neurocomputing* **138**, 69-77 (2014).

41. Azimi SM, Miar-Naimi H. Designing programmable current-mode Gaussian and bell-shaped membership function. *Analog Integrated Circuits and Signal Processing* **102**, 323-330 (2019).

42. Alimisis V, Gourdouparis M, Dimas C, Sotiriadis PP. A 0.6 V, 3.3 nW, Adjustable Gaussian Circuit for Tunable Kernel Functions. In: *2021 34th SBC/SBMicro/IEEE/ACM Symposium on Integrated Circuits and Systems Design (SBCCI)*) (2021).

43. Alimisis V, Gennis G, Touloupas K, Dimas C, Uzunoglu N, Sotiriadis PP. Nanopower Integrated Gaussian Mixture Model Classifier for Epileptic Seizure Prediction. *Bioengineering* **9**, 160 (2022).

44. Gkaltemis M-A, Lymperakis G, Gennis G, Alimisis V, Sotiriadis PP. A Hardware-Friendly Low-Power Area-Efficient GMM-Based Analog Classifier For Skin Detection. In: *2022 Panhellenic Conference on Electronics & Telecommunications (PACET)*). IEEE (2022).
